# No Faith in Physic

**Published:** 1892-01-30

**Authors:** 


### THE DOCTOR'S ARMCHAIR.

NO FAITH IN PHYSIC.

MANY people profess they have no faith in physic. The scepticism is by no means confined to non-professional persons: a certain number of doctors openly or covertly declare their unbelief, though these are not so numerous as they were a few years ago, when an airy and inconsequent scepticism was the fashionable "fad" of the day. The three successive epidemics of influenza which we have experienced during the past three winters have given point to the sarcasms of the unbelieving, and discouragement to more timid doubters. It is well worth while, therefore, at the present moment to consider the question briefly, but with the serious gravity which from every point of view it undoubtedly demands.

In the first place, one would like to suggest that faith of any sort, if it be worthy of the name, is founded either in reason or in experience. Now, most people have neither rational knowledge of physic nor personal experience. It is difficult to imagine, therefore, how such persons can expect to have any faith in it. For a man who has neither rational knowledge of a subject, nor personal experience in it, to proclaim a decided scepticism regarding it is to proclaim himself at the same time certainly unscientific, and probably a fool. It is impossible to discuss any question whatever with a person who begins the discussion by affirming his absolute disbelief in the whole business. The man who is neither a doctor himself, nor has ever been under the care of a doctor, has no more right to hold or express disbelief in physic than he has to hold or express disbelief in the existence of the great wall of China because he happens never to have seen it.

There is another class of unbelievers to whom one accords much more respect and sympathy. These are the men and women who have had too much experience of physic. They know to their cost what sort of creatures certain medical men are; and from their experience of one or two they judge all. It would be foolish to deny that there are two classes of doctors who make a great many medical sceptics. The one class is incapable, the other dishonest. There is no profession in which it is more easy to be both incapable and dishonest than in that of medicine. When a man buys a horse or a pair of boots he can at least see whether the horse or the boots have the ordinary characteristics of horses and boots, because he has had daily experience of horses and boots all his life. But when a man calls in a doctor and takes his advice and medicine, he may have had very little experience, or indeed no experience at all, of professional advice and medicine before; and he therefore has no standard of comparison whatever in his mind whereby to form an estimate of the value of the advice and medicine; he knows, in short, nothing at all about it. The advice may be the best possible or the worst possible; the medicine may be curative or poisonous. But as for the patient, he is in a position of absolute ignorance; he must take both advice and medicine on trust. Here is the opportunity of incompetence, and of knavery too. Human nature being what it is, nobody need be surprised that many doctors fail to cure easily curable diseases, and that many others drive a pushing trade in the sale of advice and medicines which are as worthless as worthless can be.

But is it either fair or wise to judge medicine by the worst specimens of its professors? No scientific man condemns religion because some clergymen are black sheep, or law because some lawyers are cheats. It is surely the proper thing to judge medicine itself by what it can do in the hands of its best professors, not in the hands of its worst. It would be very unfair to say of a horse, which could trot twelve miles an hour with a skilful rider on his back, that he was a bad horse because he failed to trot ten when he was ridden by a London tailor. The real character and merit of the horse can only be shown when he is mounted by the best possible rider. It is exactly so with medicine. Physic shows its true and proper merit only when it is practised by thoroughly competent men.

There are a certain number of medical practitioners who somewhat vauntingly proclaim their disbelief in physic; and some others who, though they do not boast of their scepticism, yet shrug their shoulders in a superior kind of fashion as much as to say that they "could a tale reveal," "an if they would." No doubt they could, and if the tale were honest, it would confess that the reason for their halting belief in physic was that they had never really taken the trouble to find out for themselves whether it was of any practical use or not. A great many people profess their disbelief in religion who have never tried what it was to be religious. But we submit that, according to the canons of science, no man is entitled to express his disbelief in religion until he has made personal experiments in it for a sufficient length of time. It is obvious that the very same rule applies to physic. How can a man who never tasted quinine in his life, or tried it properly upon others, know whether it can reduce the human temperature or not, or whether it can produce the feeling of hunger in a person who has lost his appetite. But there are at least a thousand different drugs in daily use, of which quinine is one of the commonest. If the unbelieving doctor has not been at the pains to convince himself of the beneficial action of so familiar a drug as quinine, how is it likely that he can be an authority on the hundreds of other drugs which are so much less commonly employed in practice?

The real fact is, that medicine is an exceedingly important art, and becomes more and more important as populations become more crowded, and the problems of civilization more complex. It is not now an art founded upon mere experience; it is established upon science too. Diseases in all their grosser forms are thoroughly well understood, and the physiological actions of remedies have been experimentally ascertained. For example, every medical tyro knows that opium, among its other actions, contracts the pupil and that belladonna dilates it; that Epsom salts is a purgative and that lime water constipates; that ammonia is a stimulant and potash a depressant; that strophanthus strengthens the action of the heart and that aconite weakens it; and so on *ad infinitum*. But surely if we have this amount of actual scientific knowledge, a very moderate measure of experience and care will enable us to use it to the advantage of our fellow creatures! One might as well say that Mr. Stanley would be of no more value as the leader of an African expedition than an ordinary London cabman, as say that an honest and fairly intelligent doctor can render no real service to his fellow creatures. The truth is that there is no profession which is more necessary to the public and more truly useful than that of physic, and there is no profession which is in itself more honest or more deserving of confidence. Scientific doctors play a great part in an old and advanced civilisation, and they are destined to play a still greater part as culture and knowledge increase.

